# Identification and validation of key autophagy-related genes in lupus nephritis by bioinformatics and machine learning

**DOI:** 10.1371/journal.pone.0318280

**Published:** 2025-01-27

**Authors:** Su Zhang, Weitao Hu, Yelin Tang, Xiaoqing Chen

**Affiliations:** 1 Department of Rheumatology, The Second Affiliated Hospital of Fujian Medical University, Quanzhou, P.R. China; 2 General Hospital of Ningxia Medical University, Yinchuan, P.R. China; Guangdong Nephrotic Drug Engineering Technology Research Center, Institute of Consun Co. for Chinese Medicine in Kidney Diseases, CHINA

## Abstract

**Introduction:**

Lupus nephritis (LN) is one of the most frequent and serious organic manifestations of systemic lupus erythematosus (SLE). Autophagy, a new form of programmed cell death, has been implicated in a variety of renal diseases, but the relationship between autophagy and LN remains unelucidated.

**Methods:**

We analyzed differentially expressed genes (DEGs) in kidney tissues from 14 LN patients and 7 normal controls using the GSE112943 dataset. Key modules and their contained genes were identified utilizing weighted gene co-expression network analysis (WGCNA). Differentially expressed autophagy-related genes (DE-ARGs) among DEGs, key module genes and autophagy-related genes (ARGs) were obtained by venn plot, and subjected to protein-protein interaction network construction. Two machine learning methods were applied to identify signature genes. The area under the receiver operating characteristic (ROC) curves was used to assess the accuracy of the signature genes. We also analyzed immune cell infiltration in LN. Additionally, the association between key genes and kidney diseases was predicted. Finally, key genes expression in kidney was verified by clinical samples and animal experiments.

**Results:**

A total of 10304 DEGs were identified in GSE1129943 and 29 modules were identified in WGCNA. Among them, the brown module and coral 2 module exhibited significant correlation with LN (cor = 0.86, -0.84, p<0.001). Machine learning techniques identified 5 signature genes, but only 2 were validated in the external dataset GSE32591, namely MAP1LC3B (AUC = 0.920) and TNFSF10 (AUC = 0.937), which are involved in autophagy and apoptosis. Immune infiltration analysis suggested that these key genes may be associated with immune cell infiltration in LN. In addition, these genes have been linked to a variety of renal diseases, and their expression was verified in kidney tissues in LN patients and lupus mice.

**Conclusion:**

MAP1LC3B and TNFSF10 may be key autophagy-related genes in LN. These key genes have the potential to provide new insights into the molecular diagnosis and treatment of LN.

## 1 Introduction

Lupus nephritis (LN) is a common and serious comorbidity of systemic lupus erythematosus (SLE) [1]. During the previous decades, as the recognition of the mechanisms and features of LN pathogenesis has greatly improved, more knowledge and more targeted treatment options have become available. Nevertheless, LN is still the major cause of morbidity and mortality in SLE patients [2, 3]. Currently, LN is widely accepted to control inflammation and autoimmunity mainly through corticosteroids, mycophenolate mofetil, cyclophosphamide and other immunosuppressants [4, 5]. These drugs are partially effective, but their adverse effects should not be ignored, especially with long-term use [6]. Thus, new treatments are urgently required.

Cell death is thought to be an important mechanism in the development of LN [7]. More and more evidence indicated that programmed cell death (PCD), including apoptosis, necrosis, NETosis and the response of immune are involved in the development of LN [8, 9]. Autophagy, a novel type of PCD, has attracted more and more attention in recent years. Autophagy is a self-protection mechanism of eukaryotic cells. It has an essential function in cell survival and maintenance by removing damaged cellular structures, some large molecules, and the recycling decomposition products [10]. In addition, there is accumulating evidence that dysfunctional autophagy has linked to a number of human diseases, including infection by pathogens, tumors, aging and autoimmune diseases (e.g. SLE) [11, 12]. Multiple studies support the presence of dysregulated mitochondrial autophagy in multiple cells of the blood and kidney in patients with SLE or LN and in animal models [13–16]. It has been shown that AMP-actived protein kinase (AMPK) and mammalian target of rapamycin (mTOR) are two important kinases that induce and regulate autophagy, and affect autophagy by altering the activity of Unc-51-like autophagy activating kinase (ULK), thus affecting the progression of kidney disease [17–20]. Peng et al. found that ATG5-mediated autophagy can inhibit NF-κB activation thereby limiting the inflammatory reaction in the renal epithelium, suggesting that manipulating autophagy may serve as a novel therapy for treating inflammation-induced renal disease [21]. Furthermore, Jin et al. found that UMI-77-induced mitochondrial autophagy attenuated renal fibrosis in a mouse model of unilateral ureteral obstruction [22]. All of the above evidence indicates that autophagy has a close relationship in the pathogenesis of SLE or LN. However, the function of autophagy in LN remains largely unknown and needs further investigation.

To explore whether autophagy is engaged in the development of LN, we analyzed a previously published dataset containing samples of patients with LN and non-lupus nephritis patients to determine differentially expressed genes (DEGs) related to LN. Afterwards, we further analyzed differentially expressed autophagy-related genes (DE-ARGs) and signature genes in LN by bioinformatics and machine learning methods. We also performed enrichment analysis and protein-protein interaction network (PPI) construction for DE-ARGs. In addition, we validated the diagnostic effect of signature genes in an external data set GSE32591. We also performed immune infiltration analysis. Finally, the expression of the key genes was validated in kidney tissues of LN patients and lupus mice. In conclusion, we identified 2 key-ARGs in LN and revealed their relationship with immune cells infiltration. These analyses may provide new ideas for the prevention and treatment of LN.

## 2 Materials and methods

### 2.1 Data collection

Gene microarray data from LN kidney samples were seeded from the Gene Expression Omnibus (GEO; https://www.ncbi.nlm.nih.gov/geo/) [23] database. The GSE112943 [24] was on the GPL10558 platform. This dataset contains 7 individuals without lupus nephritis (healthy control group) and 14 patients with lupus nephritis (experimental group). Follow-up analyses were performed on kidney tissue samples from 21 individuals. GSE32591 [25] was based on the GPL14663 platform and contained 64 renal tissue specimens from lupus nephritis patients and 29 renal tissue specimens from the healthy population. A total of 448 autophagy-related genes were retrieved from the human autophagy database (HADb) [26], GeneCards [27] and MsigDB databases [28] for subsequent analysis (**S1 Fig**).

### 2.2 Pre-processing of dataset and identification of differentially expressed genes in LN

The dataset is normalized by the “limma” package [29] in R to eliminate technical differences and batch effects to ensure data consistency. Principal component analysis (PCA) [30] is utilized to remove the original correlations in the dataset and perform dimensionality reduction. And analyzed gene expression in the GSE112943 dataset and filters for DEGs in LN. In brief, the genes with |log2 fold change (FC)|>0.585 and p<0.05 were considered as DEGs.

### 2.3 Identification of key modules and contained genes

The “WGCNA” package [31] of R constructed the WGCNA network on the dataset. The optimal soft threshold is determined by the picksoftThreshold function. Subsequently, TOM matrix calculation and modular clustering were performed, and modules with distances less than 0.25 were merged. Each module included at least 30 genes, and grey module was meaningless modules. In addition, the correlation coefficient between each module and LN was calculated to identify the module with the strongest correlation with LN. Finally, the correlation of key modules with LN was assessed by gene significance (GS) and the value of module memberships (MM).

### 2.4 Acquisition of differentially expressed autophagy-related genes in LN

The intersecting genes of DEGs, key module genes, and ARGs were obtained by utilizing the venn plot, namely DE-ARGs in LN.

### 2.5 Construction of protein-protein interaction networks

To understand the interactions and linkages of these DE-ARGs, the DE-ARGs were uploaded to the STRING database (https://cn.string-db.org) [32] to map their PPI networks, which were visualized by Cytoscape (version 3.9.1) [33]. The minimum required interaction score for PPI networks is 0.4. Cytoscape’s plugin molecular complex detection (MCODE) [34] was utilized to identify the most significant clusters. The parameters of the MCODE plug-in are: MCODE score > 5, degree critical value = 2, node critical value = 0.2, maximum depth = 100, k-score = 2.

### 2.6 Enrichment analysis of DE-ARGs

Gene Ontology (GO) and Kyoto Encyclopedia of Genes and Genomes (KEGG) enrichment analyses were performed to find the biological processes and pathways of DE-ARGs. GO and KEGG enrichment analyses were performed using the "clusterProfiler" package [35] of the R, and the results were visualized by bubble plots, with P <0.05 indicates statistically significant.

### 2.7 Identification of signature genes

The least absolute shrinkage and selection operator (LASSO) regression is a statistical method used for variable selection and regularization by penalizing the coefficients [36]. LASSO analysis is implemented through the “glmnet” package [37] for R. Random forest (RF) is a machine learning algorithm based on integrated learning, which effectively filters out target variables based on the importance assessment of features [38]. It is implemented through the “randomforest” package [39] of R for gene importance ranking. The intersection of the two machine learning algorithms is the signature genes in LN.

### 2.8 ROC analysis of signature genes

For testing the accuracy of machine learning screening of signature genes, the receiver operating characteristic (ROC) curves were plotted based on the training set GSE112943, where a larger area under the curve (AUC) implies a higher accuracy. The same approach was further validated in the validation set GSE32591. Validated genes are considered key ARGs in LN.

### 2.9 Interaction of key genes and diseases

The comparative toxicogenomics database (CTD) (https://ctdbase.org) [40] contains data on genes-diseases interactions. To investigate the relationship between key genes and diseases, the CTD was analyzed for inference scores and reference counts for key genes and associated diseases, and visualized by histograms.

### 2.10 Enrichment analysis of key genes

The genes most closely related to key genes were obtained from the STRING database (https://cn.string-db.org) [32]. These genes were analyzed for GO and KEGG enrichment to understand the biological processes and pathways in which they collectively participate.

### 2.11 Experiments on renal specimens

We collected paraffin sections of type IV LN and renal carcinoma paracarcinoma tissues on 1/6/2024 from the Second Affiliated Hospital of Fujian Medical University. All of the above patients were diagnosed from 1/1/2023 to 3/1/2024 by pathology. Sections were stained using rabbit anti-MAP1LC3B antibody (1:1000, 14600-1-AP, proteintech) and rabbit anti-TNFSF10 antibody (1:1000, PA5-81084, thermofisher) and incubated overnight at 4°C. Subsequently, a HRP-conjugated anti-rabbit secondary antibody was added and incubated at room temperature for 20 minutes. After that, DAB chromogen and hematoxylin counterstaining were performed. Finally, mounting treatment was conducted, and the samples were observed under a microscope.

The study was approved by the Ethics Committee of the Second Affiliated Hospital of Fujian Medical University, and all studies were conducted in accordance with relevant guidelines/regulations. Informed consent was also obtained from the patients or their families, and the ethical approval number was [2024 (337)].

### 2.12 Immune infiltration analysis

Expression profiles of GSE112943 were uploaded to the CIBERSORT website (https://cibersortx.stanford.edu/) [41] to assess differences in the composition of 22 immune cells between LN and normal kidney tissues. In addition, spearman correlation analysis was utilized to evaluate the correlation between key ARGs and immune cells.

### 2.13 Animal experiment

#### Animal selection

Female MRL/lpr mice were used as the model of spontaneous lupus, and female C57BL/6 mice of the same week were the normal controls. The urine protein of C57BL/6 mice and MRL/lpr mice was measured weekly from 16 weeks of age, and the mice were killed at 20 weeks of age after modeling. Spleen index and perirenal lymph node index were measured.

#### Immunofluorescence (IF) staining

The expression of C3 and IgG in renal tissue samples was detected by immunofluorescence. In brief, the antigen was repaired after deparaffinization, and the corresponding primary antibody was added in a 4°C refrigerator overnight. Secondary antibodies were added the next day, followed by the addition of dye and anti-fluorescence quench sealing tablets for microscopic observation.

#### Reverse Transcription-Polymerase Chain Reaction (RT-pcr)

The mRNA levels of MAP1LC3B and TNFSF10 in mice kidneys were detected by RT-pcr using the following primers (Sangon Biotech, Shanghai, China): B-actin forward: 5’-GGCTGTATTCCCCTCCATCG-3’ and reverse 5’-CCAGTTGGTAACAATGCCATGT-3’; MAP1LC3B forward: 5’- TTATAGAGCGATACAAGGGGGAG-3’ and reverse 5’- CGCCGTCTGATTATCTTGATGAG-3’; TNFSF10 forward: 5’-ATGGTGATTTGCATAGTGCTCC and reverse 5’- ATGGTGATTTGCATAGTGCTCC-3’.

### 2.14 Statistical analysis

**Fig 1:**
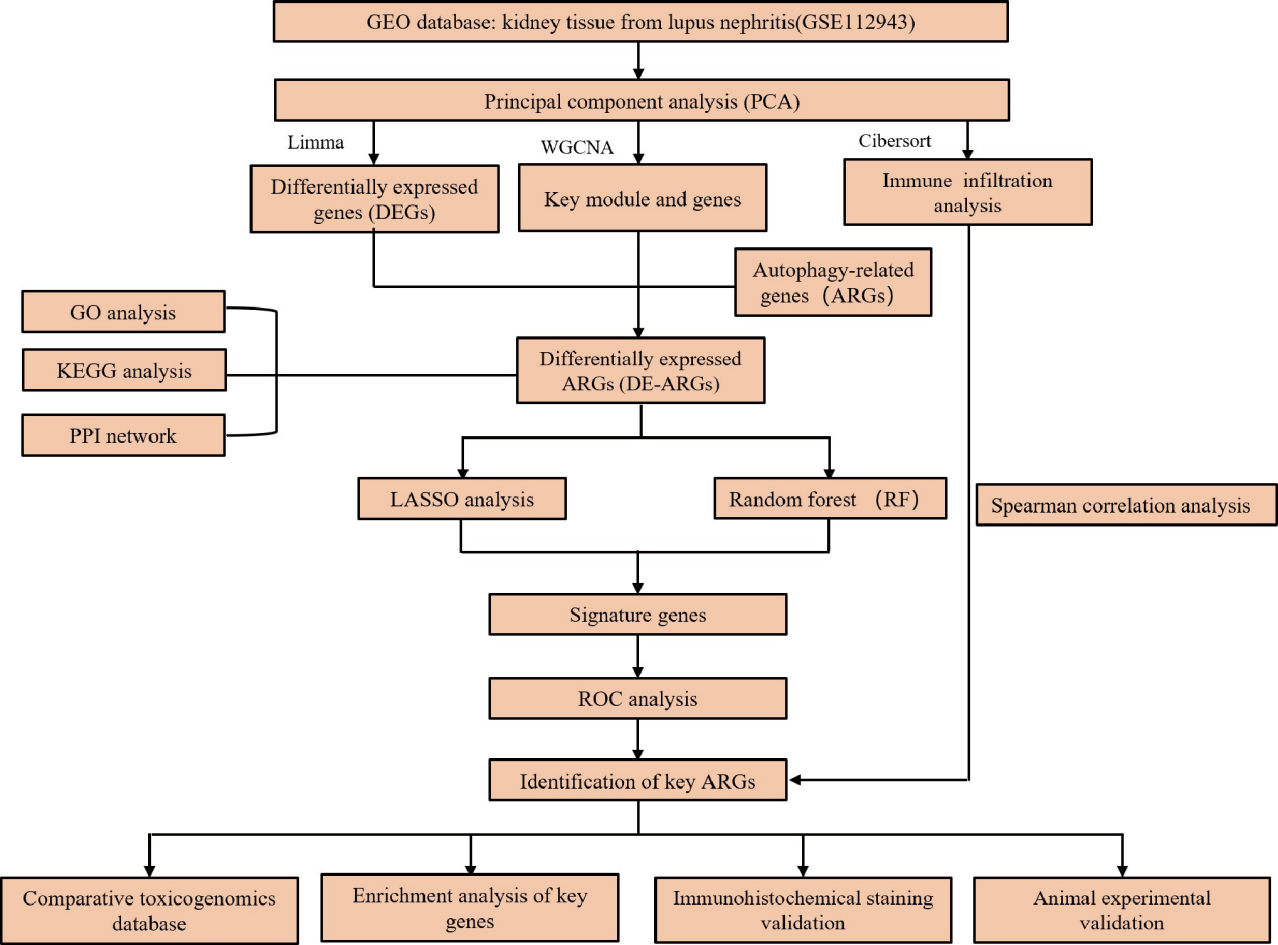
Flowchart of this study.

All analyses were conducted using R software (version 4.3.3). Spearman analysis was utilized to explore the correlation between key genes and immune cells, and p < 0.05 was regarded as statistically significant. A flowchart of the study is presented in **Fig 1**.

## 3 Results

### 3.1 Identification of DEGs

**Fig 2:**
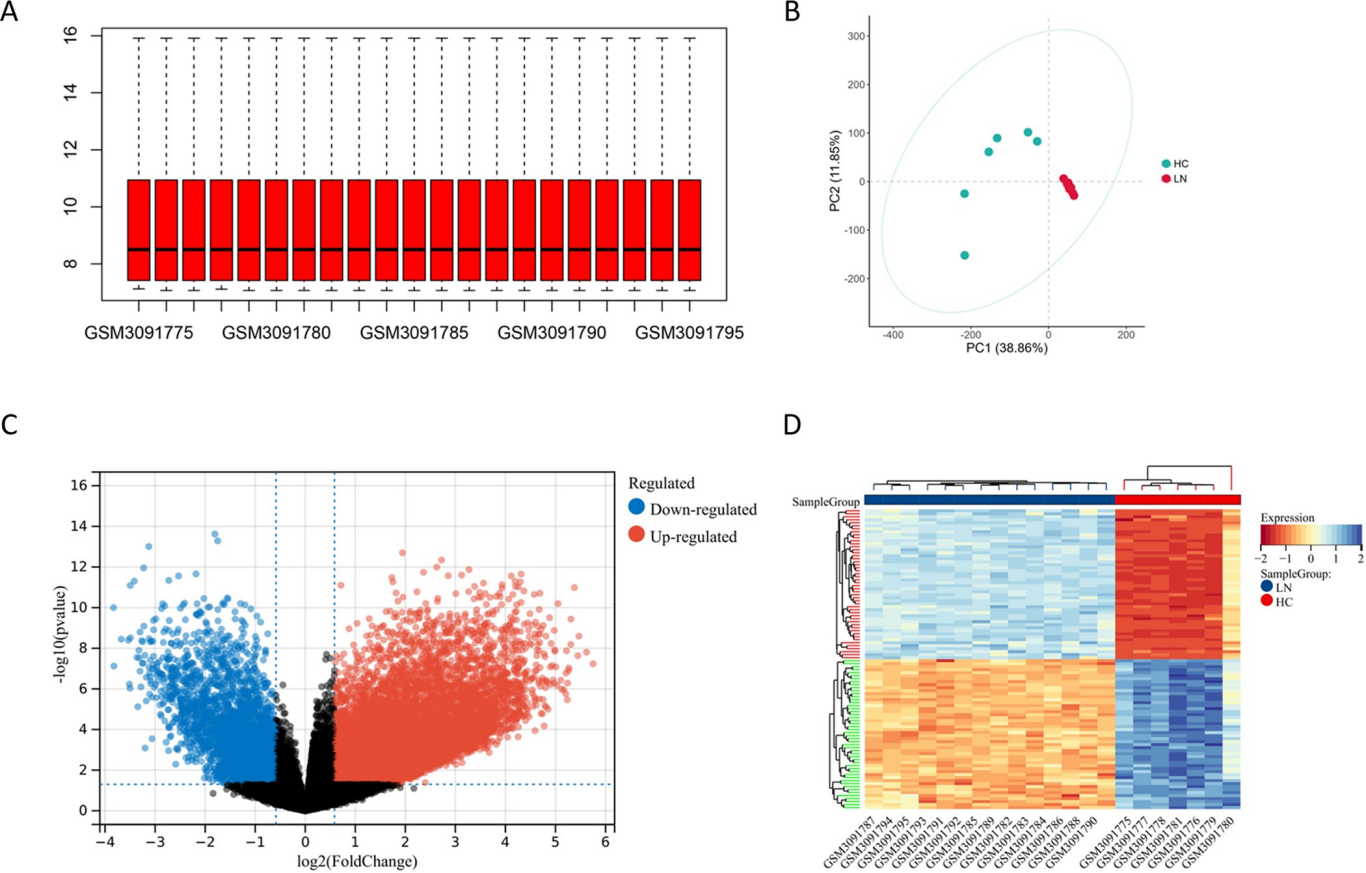
Identification of DEGs in lupus nephritis. (A) Normalization of the GSE112943 dataset. Each sample after processing is located at the same baseline. (B) PCA of the GSE112943 dataset. Elimination of different dimensional differences. (C) The volcano plot showing the expression of DEGs between lupus nephritis and normal controls. (D) The heatmap illustrating the top 50 genes with the most significant differences between individuals with lupus nephritis and normal controls.

Consistent expression levels between samples were observed after normalization of the dataset, indicating successful de-batching and facilitating subsequent studies (**Fig 2A**). After principal component analysis (PCA) of the train dataset, there were notable differences between LN patients and healthy control groups (**Fig 2B**). We identified 10304 differentially expressed genes (DEGs) in LN (**Fig 2C**). And these genes exhibited unique status in LN (**Fig 2D**).

### 3.2 The results of WGCNA of LN

**Fig 3:**
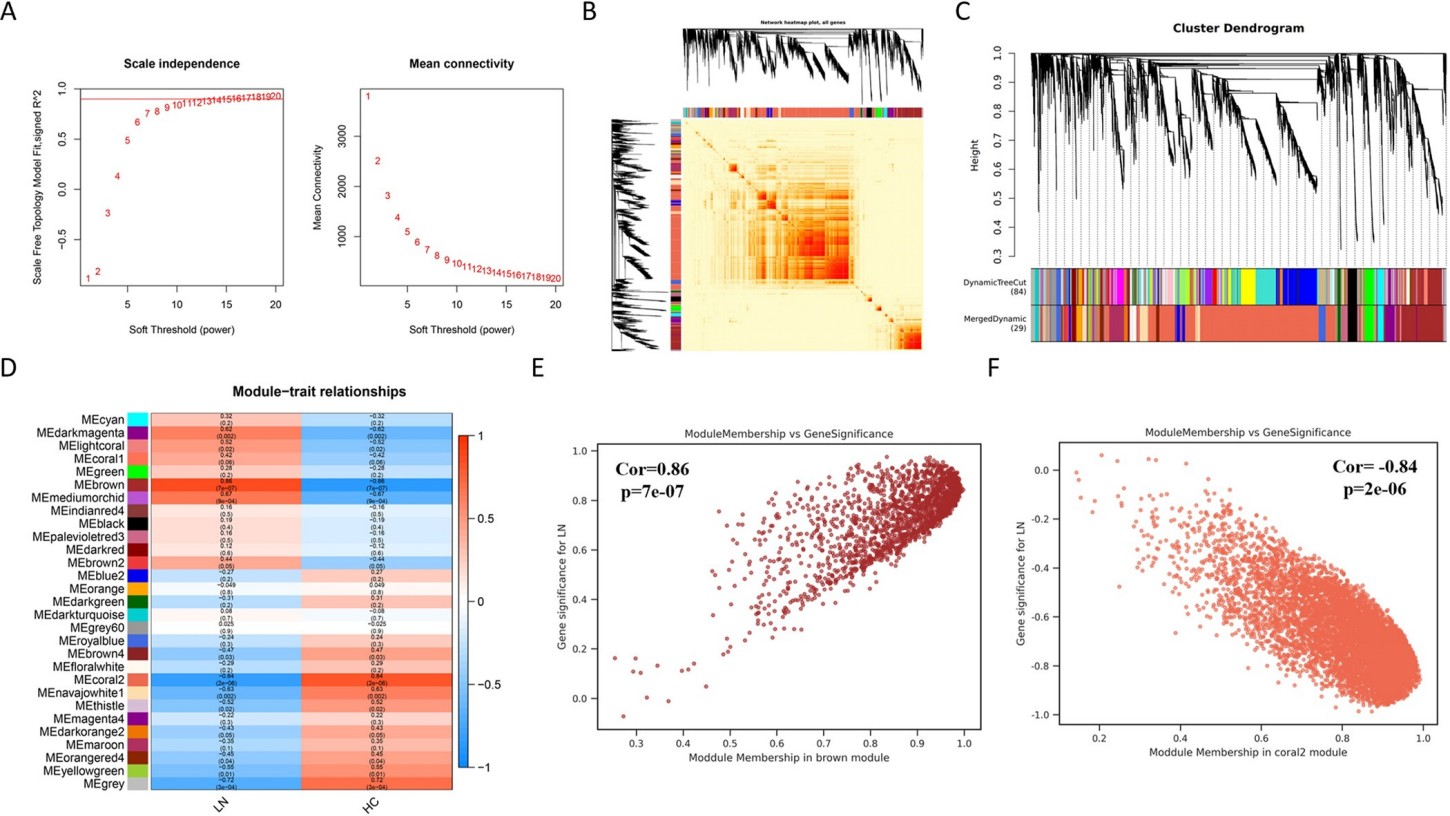
Identification of modules and genes most associated with LN based on WGCNA analysis. (A) The soft threshold power (left) and mean connectivity (right) of the WGCNA. (B) The network topology heatmap for all genes. (C) Cluster dendrogram for WGCNA analysis. (D) The heatmap showing the relationship between the modules and clinical traits, particularly lupus nephritis and normal controls. (E) The scatter plot between gene significance and module membership in the brown module. (F) The scatter plot between gene significance and module membership in the coral 2 module.

To more accurately determine the key genes associated with the LN phenotype, we performed WGCNA analysis on the training set. As shown in **Fig 3A**, the mean connectivity is optimally 0.90 when the soft threshold is 16. The heatmap of the module consisting of all genes is presented in **Fig 3B**. Through module merging, 29 gene modules with consistent co-expression trends were identified (**Fig 3C**). Among them, the “brown module”, consisting of 1733 genes, and the “coral 2 module”, consisting of 6808 genes, were most strongly correlated with LN traits (cor = 0.86, -0.84, p<0.001) (**Fig 3D** and **S2 Fig**). In addition, significant correlations between gene significance (GS) and module membership (MM) were observed within the "brown module" and the "coral 2 module", with correlation coefficients of 0.86 and -0.84, respectively, and p<0.0001 (**Fig 3E** and **3F**). The genes in these two modules were considered as key module genes.

### 3.3 Identification, PPI networks and enrichment analysis of DE-ARGs

**Fig 4:**
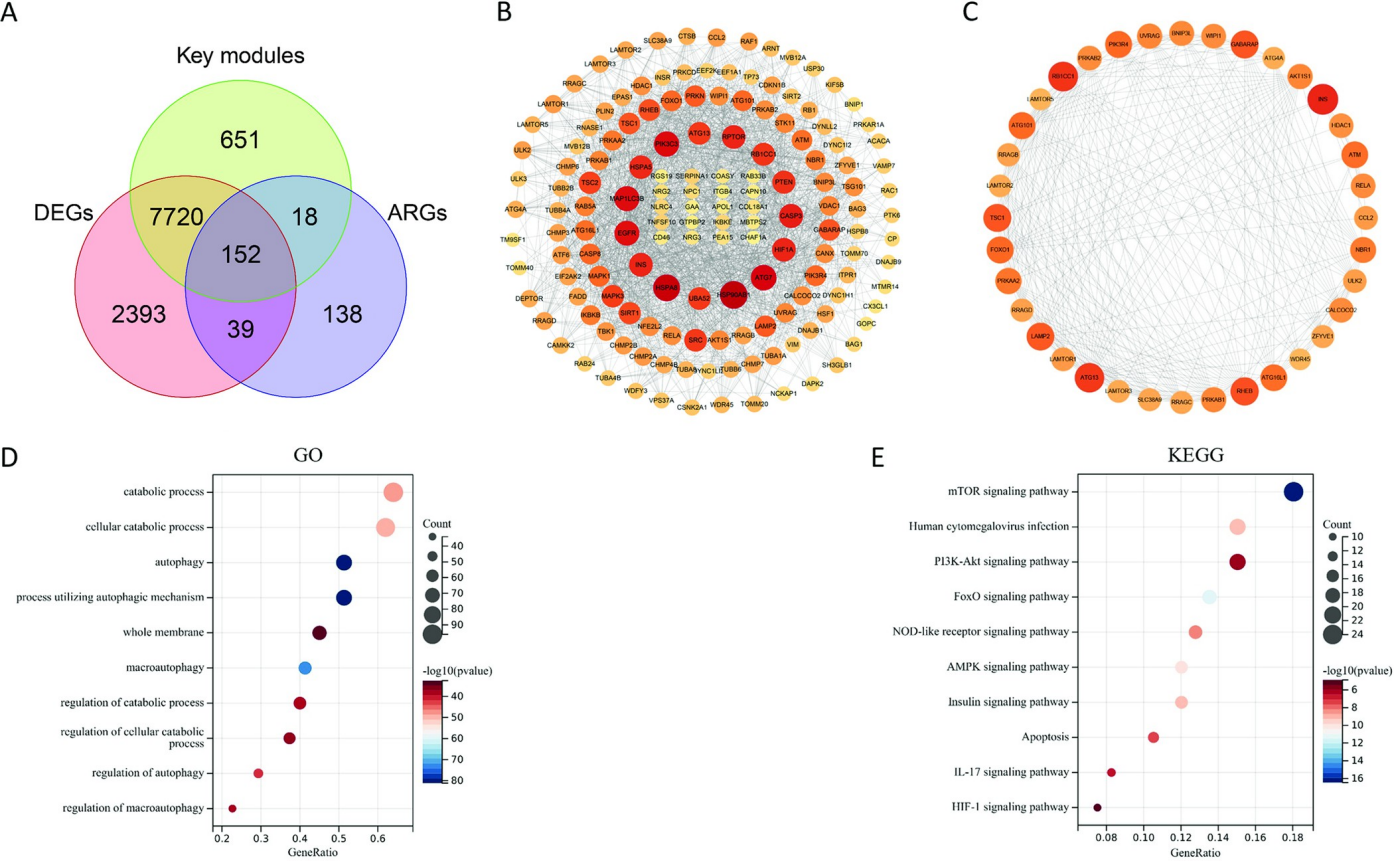
Identification of DE-ARGs. **(A) The venn diagram of the intersection of DEGs, WGCNA module genes and ARGs.** (B) The PPI network is plotted based on DE-ARGs. (C) The CytoHubba plugin filters to get the most important network clusters. (D) The bubble diagram showing the GO enrichment analysis of DE-ARGs. (E) The bubble plot displaying KEGG enrichment analysis of DE-ARGs.

To get DE-ARGs, we took the intersection of DEGs, key module genes and ARGs. As a result, a total of 152 ARGs were obtained (**Fig 4A**). After mapping the PPI network for DE-ARGs, it was found that most of them are interconnected (**Fig 4B**). The most important cluster was composed of genes such as ATG13, INS, and TSC1, which contained 36 points and 289 edges (**Fig 4C**). Through the KEGG database (https://www.genome.jp/kegg/) [42], these genes were found to be involved in the autophagy-animal pathway (**S3 Fig**).

Following GO enrichment analysis, we observed that DE-ARGs were significantly enriched in autophagy, processes utilizing autophagic mechanism, and macroautophagy (**Fig 4D**).

In regard to KEGG, we detected that DE-ARGs were mainly enriched in the mTOR signaling pathway and the FoxO signaling pathway (**Fig 4E**).

These all findings suggest that autophagy was increased and accompanied by activation of autophagy-related signaling pathways in LN patients. Meanwhile, the enrichment results were consistent with previous studies, further enhancing the credibility of this study [43, 44].

### 3.4 Identification and validation of signature genes and key ARGs

**Fig 5:**
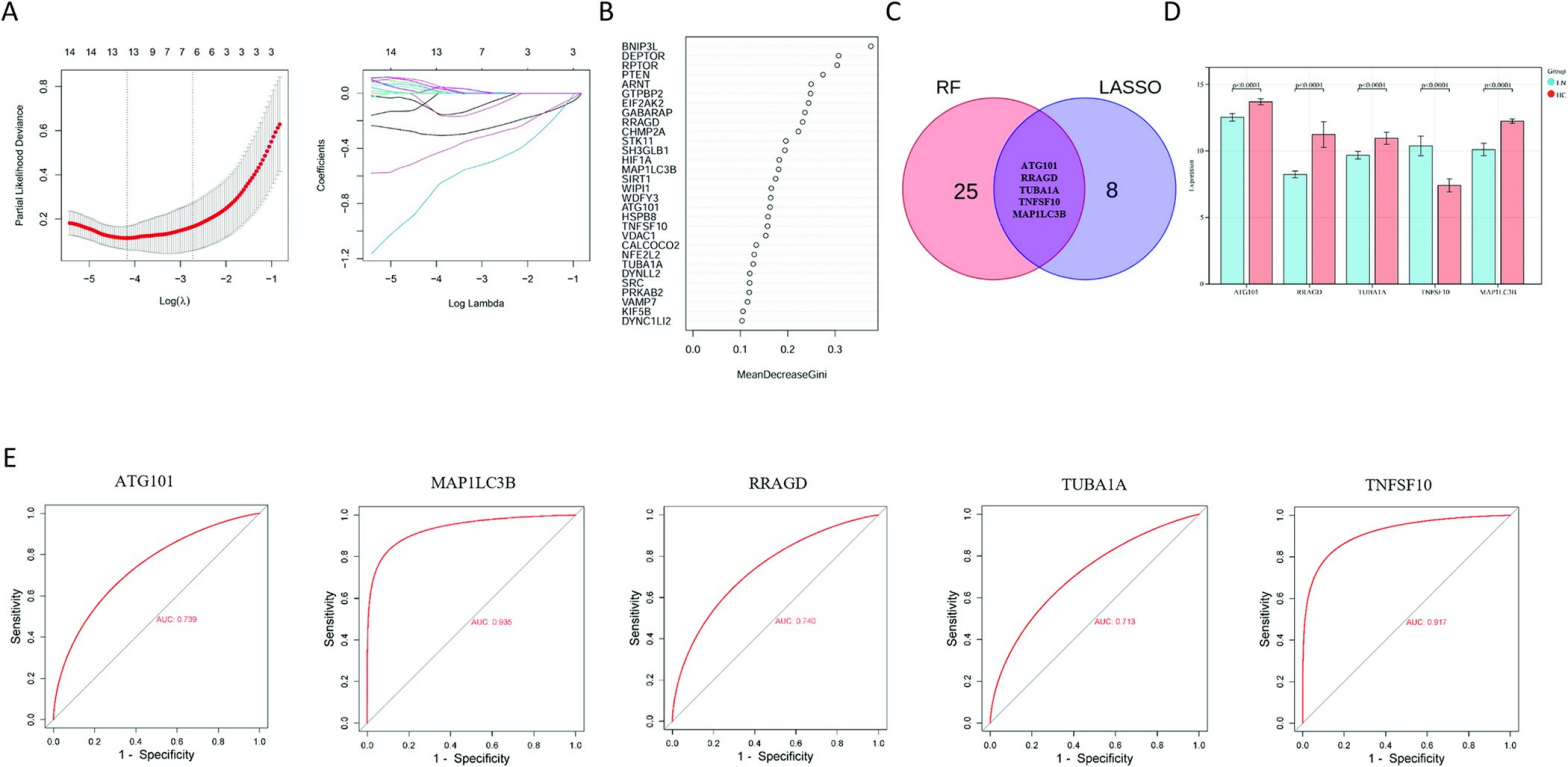
Identification of signature genes based on lasso analysis and random forests. (A) LASSO analysis of cross-validation curves. The optimal λ value was determined using 10-fold cross-validation in the retraining set (left). Penalty score plot of LASSO coefficients for signature genes associated with lupus nephritis in the training set (right). (B) The bubble plot illustrating the relative importance ranking of genes in the random forest model within the training set. (C) The venn diagram of intersection of LASSO analysis and random forest signature genes. (D) The expression levels of five signature genes in individuals with lupus nephritis in the training set compared to normal controls. (E) ROC analysis of five signature genes in the training set.

To further identify autophagy-related signature genes in LN, we applied two machine learning approaches to DE-ARGs: LASSO analysis and RF. Thirteen signature genes were ascertained by LASSO regression (**Fig 5A**), while thirty signature genes were selected by the RF algorithm (**Fig 5B**). By taking the intersection, ATG101, RRAGD, TUBA1A, MAP1LC3B and TNFSF10 were detected as common genes in both (**Fig 5C**), which are the autophagy-related signature genes in LN.

To assess the accuracy of the signature genes, we analyzed their expression levels in the training set GSE112943. We noticed that TNFSF10 was significantly up-regulated in LN compared to HC, while ATG101, RRAGD, TUBA1A, MAP1LC3B were all significantly down-regulated (**Fig 5D**). We also computed the area under the receiver operating characteristic curves (AUC-ROC) for each signature gene, which resulted in values of 0.739 for ATG101, 0.935 for MAP1LC3B, 0.740 for RRAGD, 0.713 for TUBA1A, and 0.917 for TNFSF10 (**Fig 5E**).

**Fig 6:**
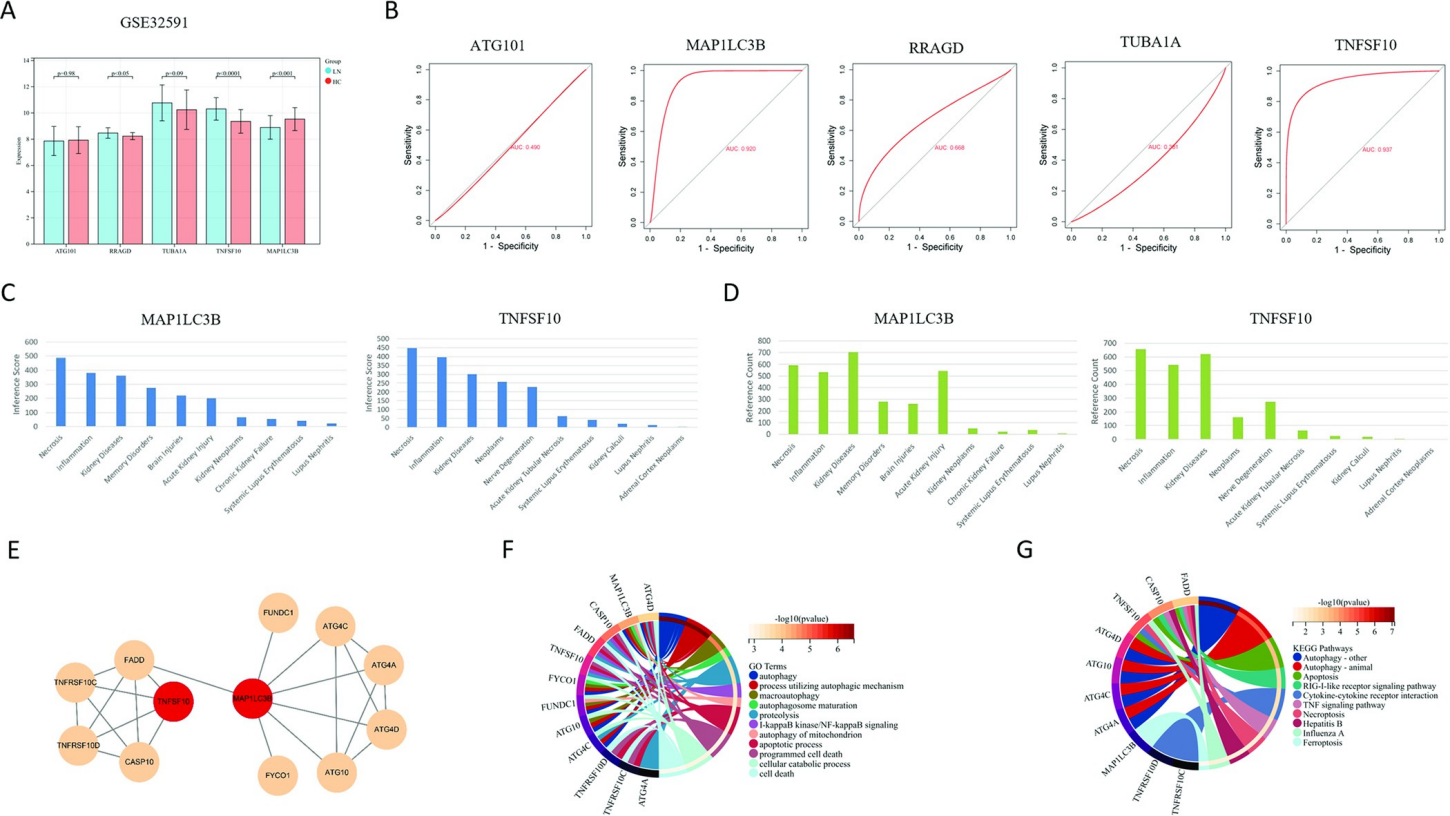
Identification of key autophagy-related genes in lupus nephritis. (A) The expression levels of five signature genes in individuals with lupus nephritis in the validating set compared to normal controls. (B) ROC analysis of five signature genes in the validating set. Inference scores (C) and reference counts (D) between key genes and necrosis, inflammation, kidney disease, memory disorders, acute kidney injury, kidney neoplasms, chronic kidney failure, systemic lupus erythematosus, lupus nephritis, and kidney calculi in the CTD database. MAP1LC3B on the left and TNFSF10 on the right. (E) Other genes that are closely related to the key genes. (F) GO enrichment analysis of key genes and their related genes. (G) KEGG enrichment analysis of key genes and their related genes.

In addition, we evaluated the expression levels and diagnostic value of the signature genes in the validation set GSE32591. We observed differences in RRAGD, MAP1LC3B and TNFSF10, and the expression of MAP1LC3B and TNFSF10 was consistent with the training set (**Fig 6A**). The AUC-ROC values in the validation set were 0.490 for ATG101, 0.920 for MAP1LC3B, 0.668 for RRAGD, 0.381 for TUBA1A and 0.937 for TNFSF10 (**Fig 6B**). Therefore, MAP1LC3B and TNFSF10 were identified as key ARGs in LN with good diagnostic value. Through a CTD database search, we observed that key ARGs were associated with a variety of kidney diseases, including LN (**Fig 6C and 6D**).

### 3.5 Functional enrichment analysis of key ARGs in LN

To clarify the biological processes and pathways of key ARGs in LN, we searched the STRING database for genes most closely related to MAP1LC3B and TNFSF10. The results revealed 10 genes closely associated with key ARGs that were connected to each other to form a network (**Fig 6E**). They were described in detail in **S4 Fig**. GO enrichment analysis of these genes was significantly enriched in autophagy (**Fig 6F**). As for KEGG analysis, they were primarily enriched in autophagy-other and autophagy-animal pathways (**Fig 6G**). These findings suggested that key genes are closely related to autophagy in LN.

### 3.6 The results of IHC staining

**Fig 7:**
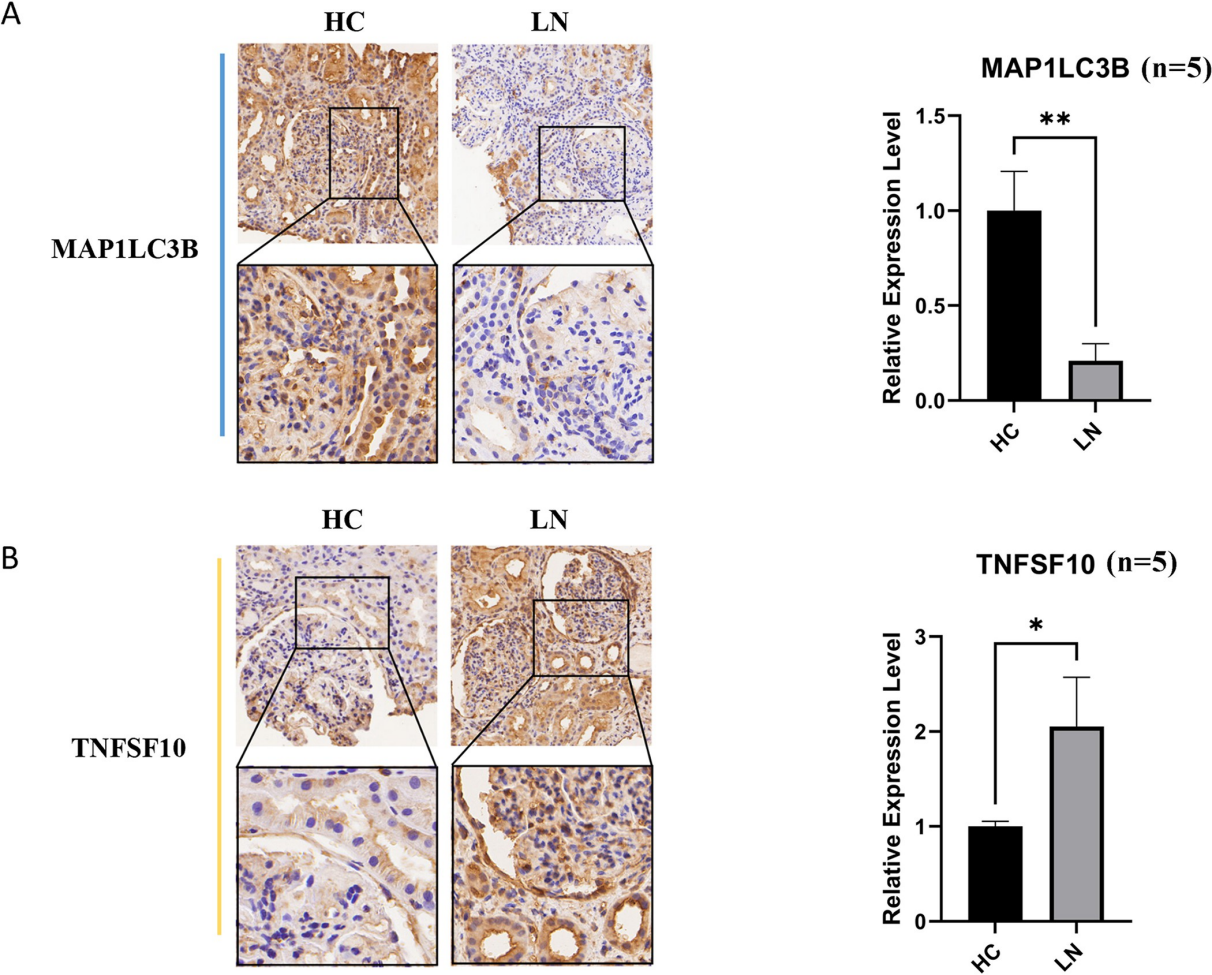
The results of immunohistochemical staining of key genes in the kidneys of patients with lupus nephritis and normal controls. (A) MAP1LC3B staining results in lupus nephritis and normal control localized renal tissues. (B) TNFSF10 staining results in lupus nephritis and normal control localized renal tissues.

To further verify the expression levels of key genes in LN patients, immunohistochemistry staining was conducted in paraffin sections of LN kidney punctures. We noted that the expression level of MAP1LC3B was significantly lower in LN than in HC (p<0.01) (**Fig 7A**), whereas TNFSF10 was significantly higher in HC (p<0.05) (**Fig 7B**). The observations were consistent with the results of bioinformatics analysis and machine learning algorithms, adding to the credibility of this study.

### 3.7 Immune infiltration analysis

**Fig 8:**
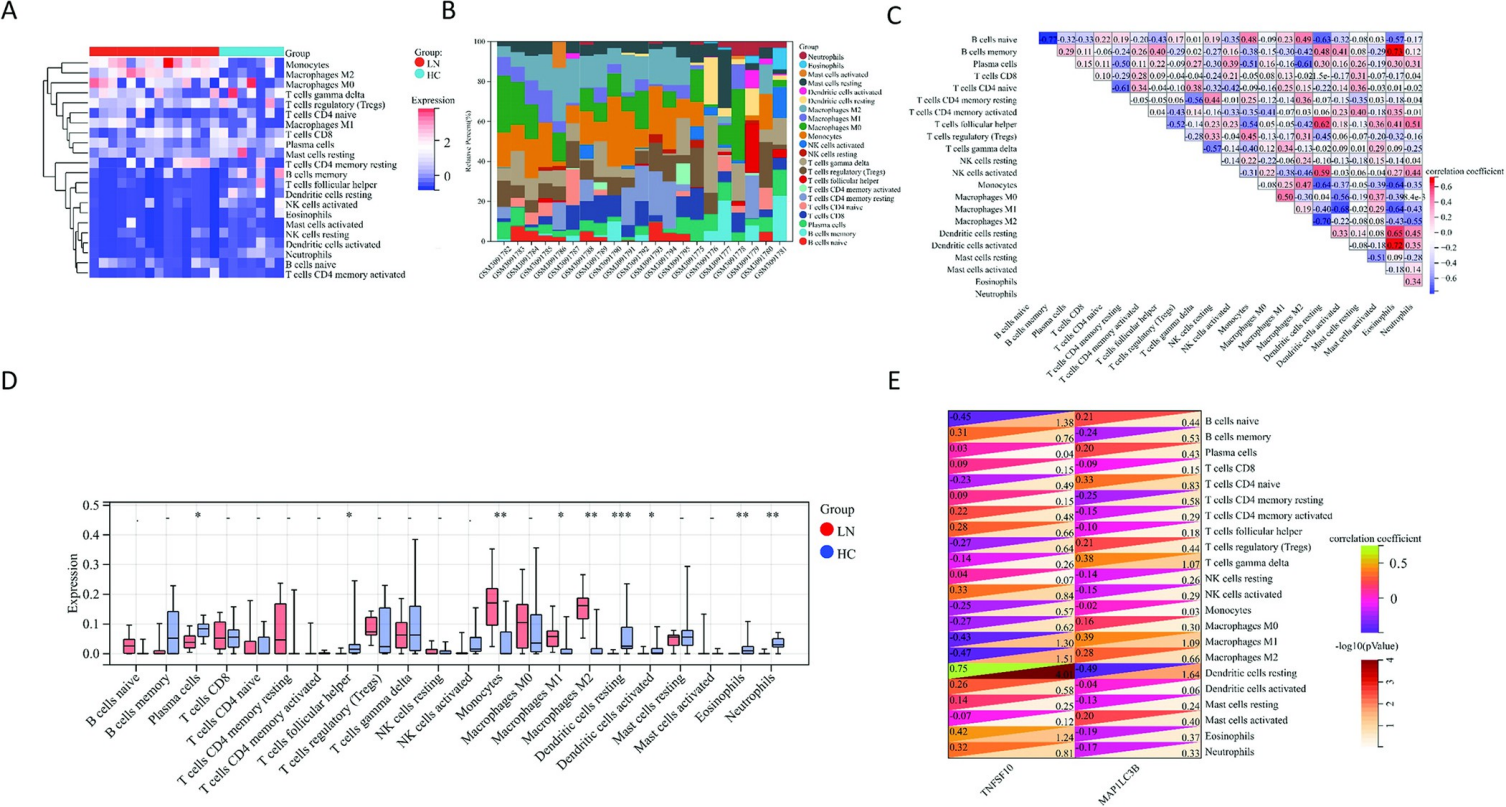
The analysis of immune infiltration in lupus nephritis and normal controls. (A) The heatmap showing the infiltration of 22 immune cells in lupus nephritis and normal control renal tissues. (B) The stacked bar graphs illustrating the composition of each immune cell in lupus nephritis and normal control renal tissues. (C) The heatmap displaying the correlation between the 22 immune cells. (D) The box plots depicting the differences in the expression of 22 immune cells between lupus nephritis and normal controls. (E) The heatmap presenting the correlation between key genes and 22 immune cells. The green color represents a positive correlation and the purple color represents a negative correlation. The darker the color, the stronger the correlation.

In renal tissues, we noted an obvious difference in immune cell infiltration between LN and HC (**Fig 8A and 8B**). In detail, monocytes and macrophages M2 were the major infiltrating cells in LN and there was a correlation between these immune cells (**Fig 8C**). Monocytes (p<0.01), macrophages M1 (p<0.05) and macrophages M2 (p<0.01) infiltration were significantly increased in LN patients. On the other hand, infiltration of plasma cells (p<0.05), T cells follicular helper (p<0.05), dendritic cells resting (p<0.001), dendritic cells activated (p<0.05), eosinophils (p<0.01), and neutrophils (p<0.01) was significantly increased in HC (**Fig 8D**). Spearman correlation analysis of key genes with immune cells indicated that TNFSF10 was positively correlated with dendritic cells resting (cor = 0.75, p<0.05) (**Fig 8E**), while MAP1LC3B was negatively correlated with dendritic cells resting (cor = -0.49, p<0.05) (**Fig 8E**). Overall, the above results indicated the association of key genes with immune cell infiltration in LN.

### 3.8 Results of animal experiments

**Fig 9:**
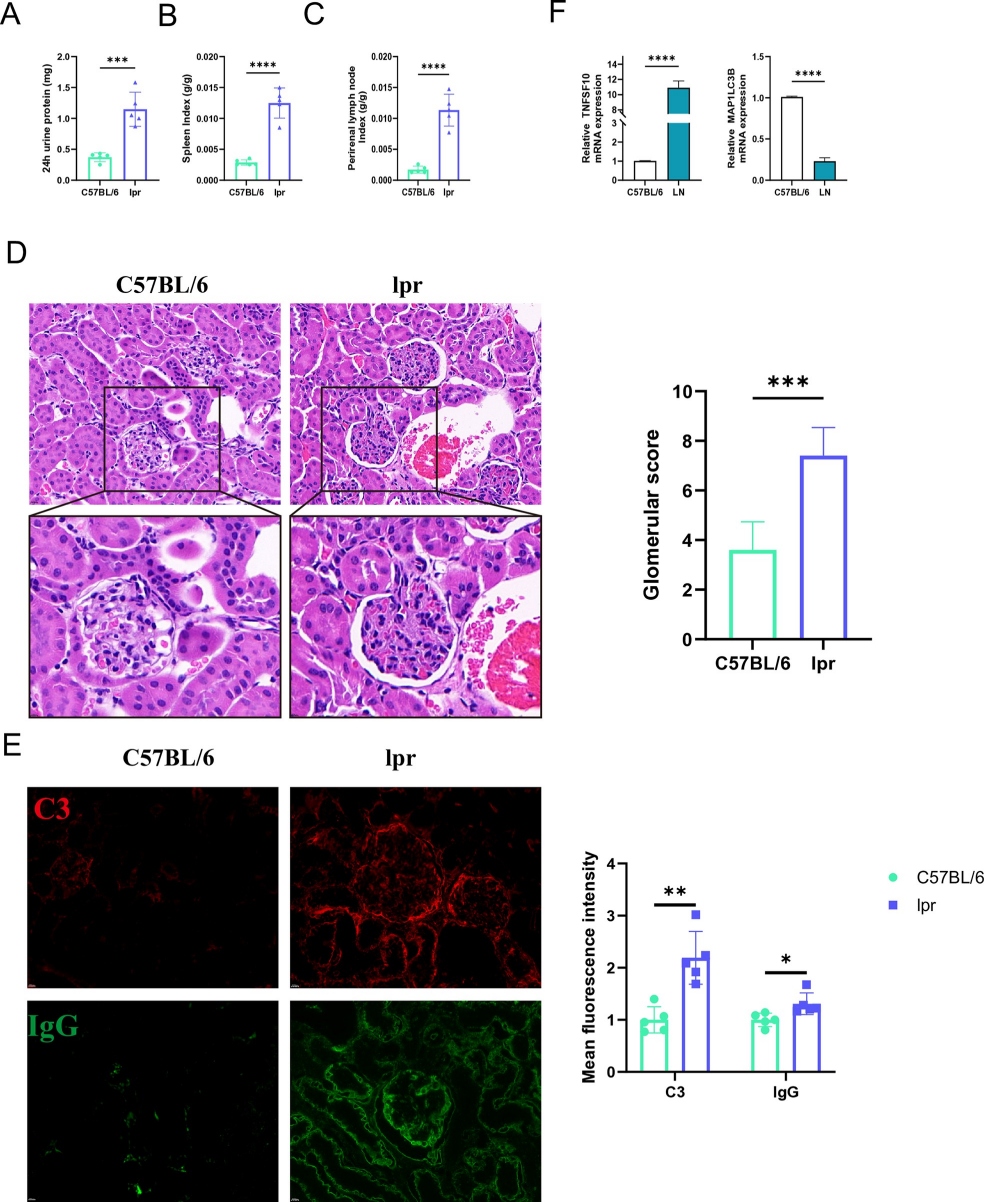
Animal experiments validate the expression of key autophagy genes. (A-C) The 24h urinary protein level, splenic index, and perirenal lymph node index in lupus mice and C57BL/6 mice. (D) HE staining and glomerular score in lupus and C57BL/6 mice. (E) C3 and IgG immunofluorescence staining in lupus mice and C57BL/6 mice. (F) The pcr results of MAPLC3B and TNFSF10.

In this study, MRL/lpr mice were used to construct lupus nephritis model, and 24h urinary protein was measured, and HE staining was used to evaluate the glomerular injury of mice. C3 and IgG immunofluorescence staining further validated the successful construction of the lupus mice model. We found that 24h urinary protein, spleen index and perirenal lymph node index of lpr mice were significantly higher than those of C57BL/6 mice (**Fig 9A–9C**). HE staining showed diffuse proliferation of mesangial cells, infiltration of inflammatory cells, and formation of interstitial fibers in severe cases of lpr mice (**Fig 9D**). Large amounts of C3 and IgG were found in the glomeruli of lpr mice by immunofluorescence. The above results confirmed the successful construction of lupus mice model (**Fig 9E**). The results of RT-pcr were also consistent with the expression trend of key ARGs in the LN dataset (**Fig 9F**).

## 4 Discussion

This study applied bioinformatics and machine learning methods to identify two key ARGs (TNFSF10 and MAP1LC3B). We observed that TNFSF10 expression was upregulated in LN, while MAP1LC3B was downregulated in LN. And the results were further validated by IHC staining. Meanwhile, these genes played a critical role in the process of autophagy. The results of the study also suggested a powerful association between key ARGs and immune cells infiltration, especially in monocytes, macrophages and dendritic cells. In addition, the present study revealed that there is a correlation between key ARGs and a variety of renal diseases, including LN. Finally, we verified the expression of TNFSF10 and MAP1LC3B in a lupus mice model. It suggests that these genes could potentially serve as biomarkers for LN.

With the development of high-throughput technology in recent years, bioinformatics approaches are increasingly playing an important role in screening DEGs in LN [45, 46]. In our study, we identified two key ARGs in LN: TNFSF10 and MAP1LC3B. Systemic lupus erythematosus (SLE) is a chronic autoimmune disease characterized by loss of self-tolerance leading to deposition of immune complexes [47]. The pathogenesis is not fully understood, and it may be the result of dysregulation of innate and adaptive immunity due to the multifactorial effects of genetics [47], endocrine [48], smoking [49] and Epstein-Barr virus infection [50]. In addition, impaired clearance of dead cells and dysregulation of antigen-presenting processes are important factors in the pathogenesis of SLE [51, 52]. Substantial evidence suggests an essential role of autophagy in the pathogenesis of SLE. Autophagy-related genes have been linked to a variety of autoimmune diseases, including SLE, through the bridge of genome-wide association studies [53]. Of these, polymorphisms in autophagy-related gene 5 (ATG5) is thought to be involved in SLE susceptibility, and clinical phenotype [54]. Moreover, aberrant expression of ATG5 can affect the removal of dead cells and antigen presentation, which may lead to SLE development [55]. The podocyte is the main constituent cell of the glomerular filtration barrier. Autoantibody and type I interferon-induced podocyte damage is one of the major causes of proteinuria in patients with LN [56]. And autophagy can attenuate the podocyte damage induced by the above mechanisms [56]. These indicate a close link between autophagy and LN.

TNFSF10, also known as TNF-related apoptotic ligand (TRAIL), is a member of the TNF ligand family. When it binds to the receptor, it can induce apoptosis [57]. TRAIL also regulates the immune response, maintains tissue homeostasis, and participates in biological processes such as inflammation, etc. The function of TRAIL proteins is important for the maintenance of normal cell survival and the regulation of the immune response [58]. There is growing evidence that TRAIL is associated with LN. A previous study reported that TRAIL expression was upregulated in renal tubular cells of patients with active LN and promoted localized renal inflammation and injury [59]. Elevated TRAIL levels were also found in the serum of adolescent lupus patients and were associated with the development and activity of nephritis [60]. Another clinical study also showed that serum TRAIL expression levels were significantly higher in patients with active lupus (SLEDAI≥4) than in healthy controls, but were not significantly elevated compared to lupus patients with SLEDAI (0–3) [61]. Various genetic studies have also revealed polymorphisms in TRAIL as a risk factor for the development of SLE [62, 63]. The failure of dead cellular debris to be eliminated in timely fashion and deposited in the kidneys is generally recognized as an essential pathogenetic mechanism of LN [64]. Dendritic cells, as important antigen-presenting cells in the body, assist the body in removing cellular debris and preventing the development of LN [65]. Our analysis showed significantly fewer dendritic cells in localized renal tissues of LN patients than in controls. It is possible that the high level of TRAIL in LN promoted apoptosis in dendritic cells.

MAP1LC3B, namely microtubule associated protein 1 light chain 3 beta (LC3B for short), is a critical marker of the autophagic process [66]. LC3B is necessary for the formation, fusion and termination processes of autophagic vesicles [67]. Autophagy plays a protective role against LN occurrence by removing dead cellular debris. LC3-associated autophagy has been recognized as the mainstay of dead cell debris removal [68]. Macrophages promote the development of SLE by releasing several inflammatory cytokines such as IFN and IL-1β [69, 70]. This is consistent with our analysis that macrophages infiltrated significantly more in the LN group than in the control group. Autophagy can reduce the production of these cytokines by macrophages, thereby limiting the inflammatory response [71]. In addition, inefficient clearance of dead cells was found in an LC3-associated autophagy-deficient mouse model, leading to increased production of inflammatory cytokines by macrophages and promoting lupus-like symptoms [72]. Yu et al. found significant differences in autophagy levels in different types of lupus nephritis. Type III, III+V and V autophagy levels were significantly more than type IV LN [73]. In summary, we considered that autophagy may play a protective role in the development of LN. Our immunohistochemical results showed that LC3B expression was down-regulated in localized renal tissues of LN, which may favor the development of LN. Of course, the specific mechanism still needs to be further explored.

Immune cell infiltration is a feature of LN. In particular, monocytes and macrophages infiltrate renal tissue and exacerbate renal injury by producing chemokines and cytokines [74–79]. Although other immune cell infiltrations, such as eosinophils and follicular helper T cells, were present in LN renal tissues, their exact relationship with LN remains unclear [80–83]. Our findings revealed that M1 macrophages, M2 macrophages and monocytes were significantly increased in LN kidney tissues. While dendritic cells were significantly decreased in LN kidney tissues. Furthermore, there is a correlation between key autophagy genes and the above mentioned immune cells, which more strongly supports the non-negligible role of immune dysregulation in the development of LN.

Our study firstly identified 1010 DEGs in LN. Two key autophagy-related genes were of particular interest in our subsequent analyses: MAP1LC3B and TNFSF10. These genes may be involved in LN through dendritic cells and macrophages. These key genes have the potential to be reliable biomarkers for LN and may also provide new ideas for the treatment of LN. Of course, further in vivo and in vitro experiments are needed to continue exploration and validation.

## Supporting information

S1 Fig Autophagy-related genes from HADb, GeneCards, and MsigDB database were searched. (PDF)

S2 Fig The correlation of each module with the phenotype and the number of genes included. Red color represents positive correlation and blue color represents negative correlation. The darker the color, the stronger the correlation. The number of genes included is in parentheses. * indicates p<0.05, ** indicates p<0.01, and *** indicates p<0.001.
(PDF)

S3 Fig Schematic diagram of the autophagy-animal signaling pathway. The figure shows that the genes constituting the most important PPI network are involved in the pathway.
(PDF)

S4 Fig Detailed information on key autophagy-related genes and the genes they are most closely related to. (PDF)

S1 Raw data (XLSX)
