# Supplementary material for: Identification and validation of key autophagy-related genes in lupus nephritis by bioinformatics and machine learning
Source: PLoS One. 2025 Jan 27;20(1):e0318280. doi: 10.1371/journal.pone.0318280 (PMC11771862; doi:10.1371/journal.pone.0318280)
Supplement: S1 Fig — (PDF) [file pone.0318280.s001.pdf]

Database	Total number of genes	Gene symbol
HADb	222	AMBRA1 APOL1 ARNT ARSA ARSB ATF4 ATF6 ATG10 ATG12 ATG16L1 ATG16L2 ATG2A ATG2B ATG3 ATG4A ATG4B ATG4C ATG4D ATG5 ATG7 ATG9A ATG9B ATIC BAG1 BAG3 BAK1 BAX BCL2 BCL2L1 BECN1 BID BIRC5 BIRC6 BNIP1 BNIP3 BNIP3L C12orf44 C17orf88 CALCOCO2 CAMKK2 CANX CAPN1 CAPN10 CAPN2 CAPNS1 CASP1 CASP3 CASP4 CASP8 CCL2 CCR2 CD46 CDKN1A CDKN1B CDKN2A CFLAR CHMP2B CHMP4B CLN3 CTSB CTSD CTSL1 CX3CL1 CXCR4 DAPK1 DAPK2 DDIT3 DIRAS3 DLC1 DNAJB1 DNAJB9 DRAM1 EDEM1 EEF2 EEF2K EGFR EIF2AK2 EIF2AK3 EIF2S1 EIF4EBP1 EIF4G1 ERBB2 ERN1 ERO1L FADD FAM48A FAS FKBP1A FKBP1B FOS FOXO1 FOXO3 GAA GABARAP GABARAPL1 GABARAPL2 GAPDH GNAI3 GNB2L1 GOPC GRID1 GRID2 HDAC1 HDAC6 HGS HIF1A HSP90AB1 HSPA5 HSPA8 HSPB8 IFNG IKBKB IKBKE IL24 IRGM ITGA3 ITGA6 ITGB1 ITGB4 ITPR1 KIAA0226 KIAA0652 KIAA0831 KIF5B KLHL24 LAMP1 LAMP2 MAP1LC3A MAP1LC3B MAP1LC3C MAP2K7 MAPK1 MAPK3 MAPK8 MAPK8IP1 MAPK9 MBTPS2 MLST8 MTMR14 MTOR MYC NAF1 NAMPT NBR1 NCKAP1 NFE2L2 NFKB1 NKX2-3 NLRC4 NPC1 NRG1 NRG2 NRG3 P4HB PARK2 PARP1 PEA15 PELP1 PEX14 PEX3 PIK3C3 PIK3R4 PINK1 PPP1R15A PRKAB1 PRKAR1A PRKCD PRKCQ PTEN PTK6 RAB11A RAB1A RAB24 RAB33B RAB5A RAB7A RAC1 RAF1 RB1 RB1CC1 RELA RGS19 RHEB RPS6KB1 RPTOR SAR1A SERPINA1 SESN2 SH3GLB1 SIRT1 SIRT2 SPHK1 SPNS1 SQSTM1 ST13 STK11 TBK1 TM9SF1 TMEM49 TMEM74 TNFSF10 TP53 TP53INP2 TP63 TP73 TSC1 TSC2 TUSC1 ULK1 ULK2 ULK3 USP10 UVRAG VAMP3 VAMP7 VEGFA WDFY3 WDR45 WDR45L WIPI1 WIPI2 ZFYVE1
GeneCards	63	ATG7 ATG10 DEPTOR ATG9A ATG2A COASY ULK1 BECN1 ATG12 MTOR PIK3R4 PIK3C3 ATG16L1 ATG3 ATG5 ATG13 RB1CC1 ATG14 WIPI1 MAP1LC3A ULK2 ATG4A WIPI2 PRKAA1AMBRA1 RPTOR ATG101 MLST8 UVRAG AKT1S1 ZFYVE1 TSC2 PRKAG2 PRKAG3 PRKAA2 PRKAB1 PRKAB2 PRKAG1 MAP1LC3B CHAF1A ATG9B NSR SH3GLB1 BCL2 VMP1 INS SPTLC1 WDR45 CP ATP13A2 FTL ACACA MECP2 GTPBP2 RHEB SCP2 STK1 FA2H PANK2 DCAF17 PLA2G6 ATG16L2
MsigDB	163	CFTR LAMP2 RB1CC1 RRAGD VIM IFT88 GABARAPL2 ATG5 FUNDC1 WIPI1 CSNK2A2 TSG101 TUBA3D DYNC1I2 PIK3C3 HSP90AA1 RRAGB VPS37B MAP1LC3B RPTOR WDR45B VDAC1 CSNK2B TUBB8 PEX5 CHMP2B ATG16L1 DYNLL1 HDAC6 HSP90AB1 TOMM22 MTMR3 TUBB1 CSNK2A1 CHMP4B MAP1LC3A ATG4A TSC2 TUBB4A PLIN3 RHEB MVB12A RPS27A DYNC1LI1 ATG3 TOMM6 TUBA4B DYNLL2 PRKAG2 LAMTOR3 HSPA8 AMBRA1 PRKAB1 CHMP3 PRKAG3 EPAS1 PARK7 LAMTOR2 MFN2 RRAGC ATG101 TUBA1B ATG4C BECN1 ATG12 CHMP7 PLIN2 ATM UBA52 UBE2V1 ATG16L1 ATG14 TUBA4A RNASE1 TOMM40 CHMP2A ATG4D GFAP PRKAB2 PRKAA1 AMTOR5 USP30 DYNC1LI2 TUBB2A TUBB2B ABARAPL1 PEX5 LAMTOR1 UBC TUBA3E ATG10 CSNK2B HBB SQSTM1 TOMM70 RRAGA VPS37A MTERF3 EEF1A1 WIPI2 DYNC1I1 PINK1 PCNT VPS28 SQSTM1 PRKAA2 MTMR14 CHMP4C UBAP1 VCP TSC1 TUBA1A TUBA1C MLST8 CSNK2B PGAM5 HSF1 VPS37C ATG4B ARL13B GABARAP UBB MFN1 TUBB8B TOMM20 ATG13 TOMM5 TUBB6 CHMP6 VPS37D SLC38A9 CETN1 ULK1 UBE2N TUBAL3 ATG9B PRKAG1 CSNK2B CHMP4A CHMP4A TUBA8 HSF1 PRKN LAMTOR4 TUBB4B NBR1 PIK3R4 TOMM7 MVB12B WDR45 DYNC1H1 SRC ATG7 MAP1LC3C TUBA3C UVRAG MTOR ATG9A CSNK2B CSNK2B CSNK2B TUBB VPS28
