# Supplementary figures and images for: Identification and validation of key autophagy-related genes in lupus nephritis by bioinformatics and machine learning
Source: PLoS One. 2025 Jan 27;20(1):e0318280. doi: 10.1371/journal.pone.0318280 (PMC11771862; doi:10.1371/journal.pone.0318280)

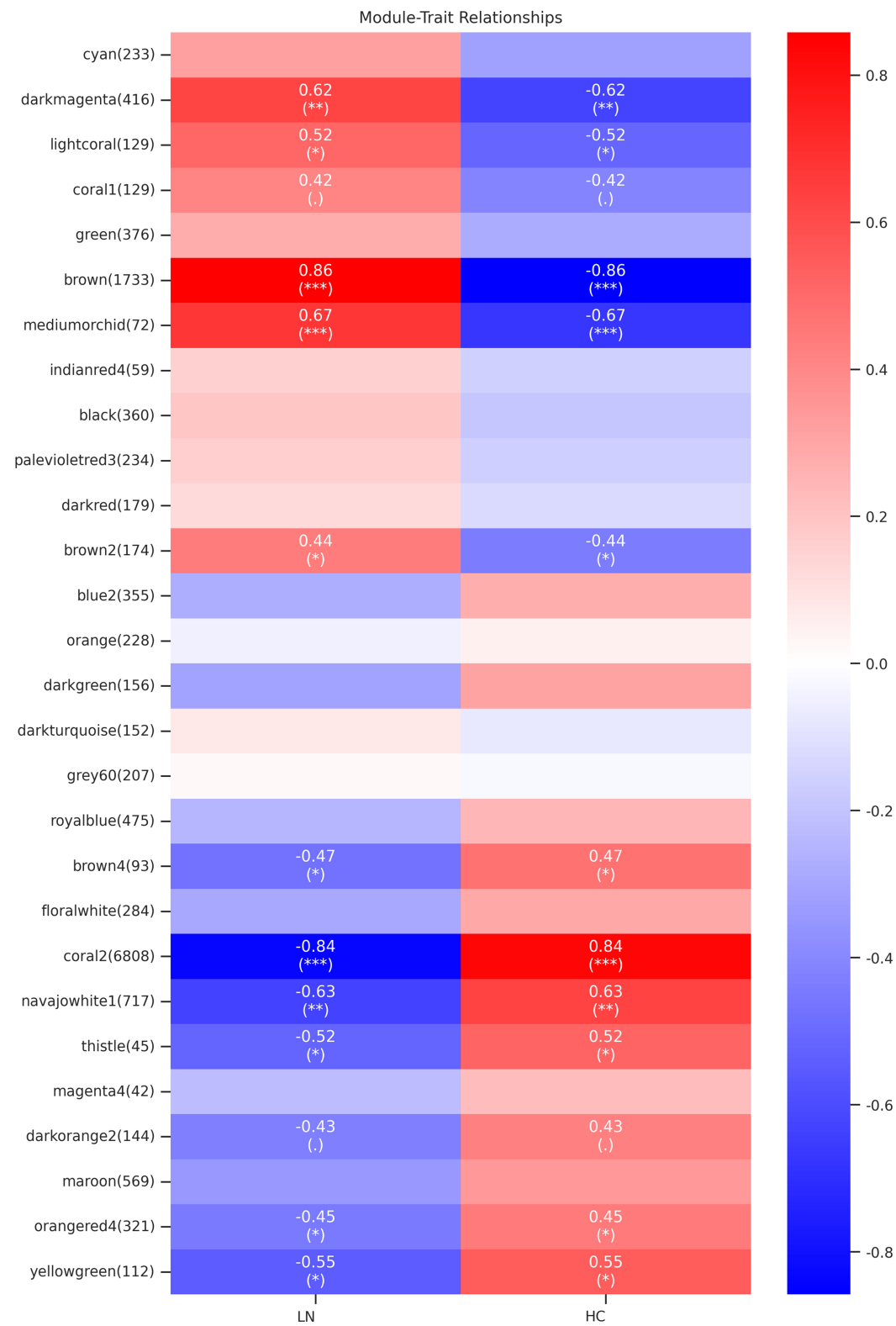

Supplement: S2 Fig — Red color represents positive correlation and blue color represents negative correlation. The darker the color, the stronger the correlation. The number of genes included is in parentheses. * indicates p<0.05, ** indicates p<0.01, and *** indicates p<0.001. (PDF) [file pone.0318280.s002.pdf]

# AUTOPHAGY - ANIMAL

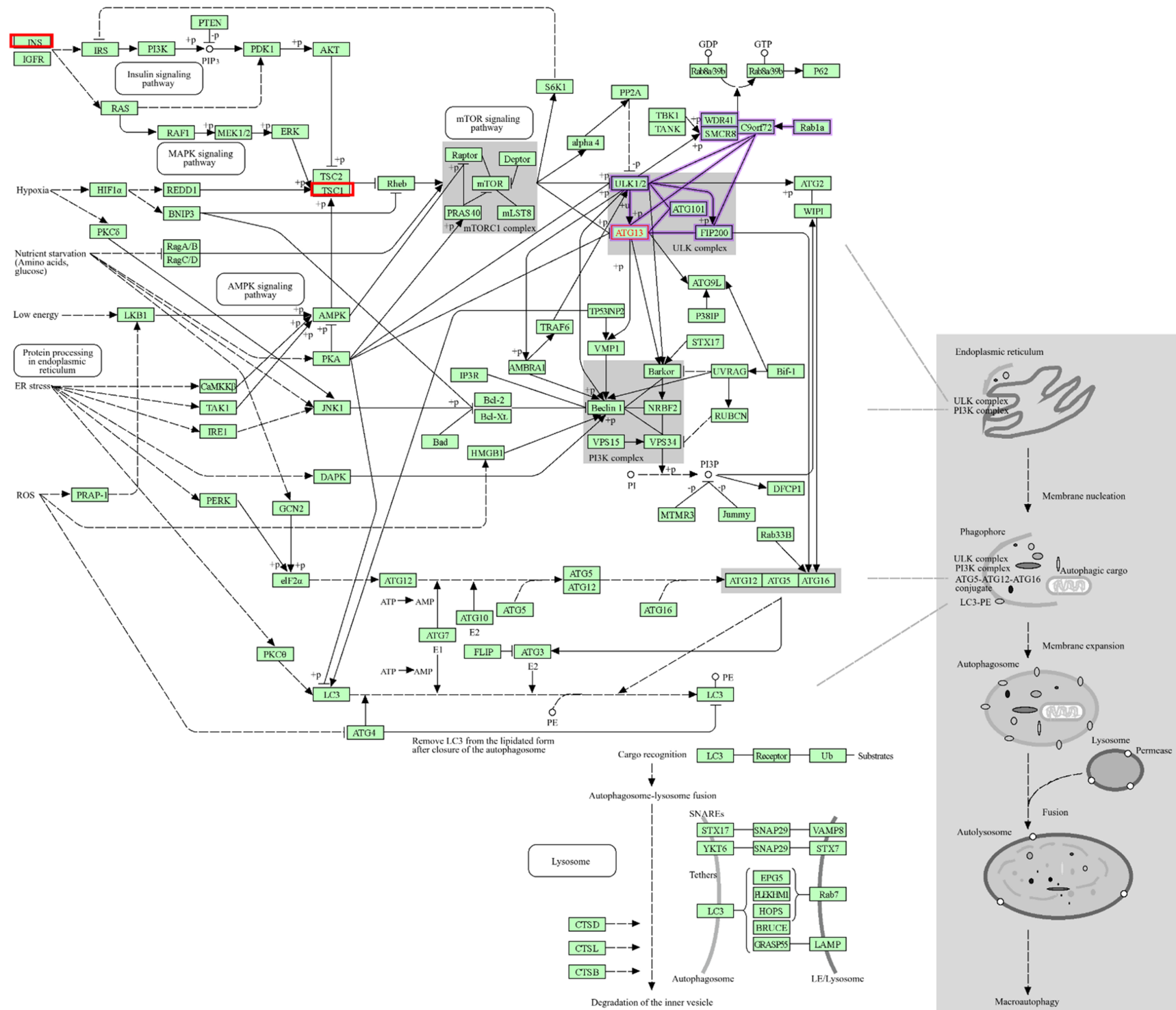

Supplement: S3 Fig — The figure shows that the genes constituting the most important PPI network are involved in the pathway. (PDF) [file pone.0318280.s003.pdf]
