# Supplementary material for: Identification and validation of key autophagy-related genes in lupus nephritis by bioinformatics and machine learning
Source: PLoS One. 2025 Jan 27;20(1):e0318280. doi: 10.1371/journal.pone.0318280 (PMC11771862; doi:10.1371/journal.pone.0318280)
Supplement: S4 Fig — (PDF) [file pone.0318280.s004.pdf]

Key autophagy genes and their related genes

Gene symbol	Description	Function
MAP1LC3B	Microtubule Associated Protein 1 Light Chain 3 Beta	Ubiquitin-like modifier involved in formation of autophagosomal vacuoles (autophagosomes). Plays a role in mitophagy which contributes to regulate mitochondrial quantity and quality by eliminating the mitochondria to a basal level to fulfill cellular energy requirements and preventing excess ROS production. Upon nutrient stress, directly recruits cofactor JMY to the phagophore membrane surfaces and promotes JMY's actin nucleation activity and autophagosome biogenesis during autophagy.
TNFSF10	TNF Receptor Superfamily Member 10	The protein encoded by this gene is a cytokine that belongs to the tumor necrosis factor (TNF) ligand family. This protein preferentially induces apoptosis in transformed and tumor cells, but does not appear to kill normal cells although it is expressed at a significant level in most normal tissues.
ATG4A	Autophagy-Related Protein 4 Homolog A	Cysteine protease that plays a key role in autophagy by mediating both proteolytic activation and delipidation of ATG8 family proteins.
ATG4C	Autophagy-Related Protein 4 Homolog C	Same as ATG4A.
ATG4D	Autophagy-Related Protein 4 Homolog D	Plays a role as an autophagy regulator that links mitochondrial dysfunction with apoptosis. The mitochondrial import of ATG4D during cellular stress and differentiation may play important roles in the regulation of mitochondrial physiology, ROS, mitophagy and cell viability.
ATG10	Autophagy-Related Protein 10	E2-like enzyme involved in autophagy. Acts as an E2-like enzyme that catalyzes the conjugation of ATG12 to ATG5. Likely serves as an ATG5-recognition molecule.
FUNDC1	FUN14 Domain-Containing Protein 1	Acts as an activator of hypoxia-induced mitophagy, an important mechanism for mitochondrial quality control.
FYCO1	FYVE And Coiled-Coil Domain Autophagy Adaptor 1	May mediate microtubule plus end-directed vesicle transport.
FADD	Fas Associated Via Death Domain	Apoptotic adaptor molecule that recruits caspase-8 or caspase-10 to the activated Fas (CD95) or TNFR-1 receptors. The resulting aggregate called the death-inducing signaling complex (DISC) performs caspase-8 proteolytic activation. Active caspase-8 initiates the subsequent cascade of caspases mediating apoptosis.
CASP10	Caspase 10	This gene encodes a protein which is a member of the cysteine-aspartic acid protease (caspase) family. Sequential activation of caspases plays a central role in the execution-phase of cell apoptosis.
TNFRSF10C	TNF Receptor Superfamily Member 10c	Receptor for the cytotoxic ligand TRAIL. Lacks a cytoplasmic death domain and hence is not capable of inducing apoptosis. May protect cells against TRAIL mediated apoptosis by competing with TRAIL-R1 and R2 for binding to the ligand.
TNFRSF10D	TNF Receptor Superfamily Member 10d	Receptor for the cytotoxic ligand TRAIL. Contains a truncated death domain and hence is not capable of inducing apoptosis but protects against TRAIL-mediated apoptosis.
